# Trophic position structures physiological trade-offs between metabolic and detoxification pathways in sympatric passerines

**DOI:** 10.1098/rsos.261499

**Published:** 2026-10-07

**Authors:** Pablo Sabat, Antonia Beltran-Hayet, Francisco del Basto, Benjamin Corvalán, Natalia Ramirez-Otarola, Felipe Alvarez-Vergara, Diego Landaeta, Roberto Nespolo, Juan C. Sanchez-Hernandez, Karin Maldonado, Seth D. Newsome

**Affiliations:** ^1^ Departamento de Ciencias Ecológicas, Universidad de Chile, Facultad de Ciencias, Ñuñoa, Santiago Metropolitan Region, Chile; ^2^ Escuela de Medicina Veterinaria, Universidad Mayor, Santiago, Chile; ^3^ Universidad Austral de Chile, Valdivia, Chile; ^4^ Instituto de Ciencias Ambientales y Evolutivas, Valdivia, Chile; ^5^ Millenium Nucleus of Patagonian Limit of Life (LiLi), Valdivia, Chile; ^6^ Department of Environmental Sciences, Universidad de Castilla-La Mancha—Campus de Toledo, Toledo, Spain; ^7^ Departamento de Ciencias, Universidad Adolfo Ibanez, Peñalolén, Santiago Metropolitan Region, Chile; ^8^ Department of Biology, University of New Mexico Consortium of the Americas for Interdisciplinary Science, Albuquerque, NM, USA

**Keywords:** birds, detoxification pathways, immune function, oxidative metabolism, trophic level, stable isotopes

## Abstract

Resource use links ecological context to organismal performance, yet whether dietary breadth or trophic position is more closely associated with physiological state in wild birds remains unclear. We used a natural trophic gradient in a sympatric passerine assemblage to test whether trophic position and isotopic niche breadth are associated with integrated physiological phenotypes. We studied three co-occurring passerine species in central Chile spanning a trophic spectrum (granivorous, omnivorous, insectivorous). Using blood δ^13^C and δ^15^N, we quantified isotopic niche width and estimated trophic position, then compared these metrics with physiological variables measured in free-living birds (*n* = 60), including differential leukocyte counts, antioxidant capacity, and enzymatic markers of oxidative metabolism, glutathione-related physiology, and plasma esterase activity. Isotopic niche width differed among species but was weakly associated with physiological variation. By contrast, estimated trophic position showed stronger species-level correspondence with multivariate physiological organization. Metabolic and enzyme-based variables contributed most to interspecific physiological differentiation, whereas baseline non-enzymatic antioxidant buffering was conserved. Overall, estimated trophic position showed a stronger correspondence with coordinated physiological variation than isotopic niche breadth. These findings highlight species-level physiological differences associated with trophic position and provide a basis for broader comparative analyses of ecological and evolutionary influences on avian physiology.

## 1. Introduction

Studying how ecological and nutritional variation shape physiological state in free-living birds can help clarify how resource use covaries with organismal performance under natural conditions [1,2]. Among the ecological factors that influence physiological function, the quantity and quality of available resources are especially important because they connect environmental conditions with energetic and physiological processes that affect performance. Variation in diet quality and composition can therefore be associated with oxidative status, immune-related traits [1,3,4], and broader physiological variation.

In birds, species differ in the nutritional and biochemical characteristics of consumed foods [5,6], which can influence digestion, metabolic processes, renal function, and oxidative balance [6–10]. Dietary differences may also be reflected in enzyme systems involved in processing dietary compounds, as diets differ not only in nutrient composition but also in their content of plant secondary metabolites [9–12].

Leukocyte profiles provide valuable insight into the physiological state of birds, reflecting variation in stress physiology, infection-related processes, and leukocyte composition [13,14]. The heterophil:lymphocyte (H:L) ratio is widely used as an indicator of physiological stress and is most informative when interpreted in relation to leukocyte redistribution among immune cell classes [13,15]. Similarly, total leukocyte counts provide relevant information regarding physiological condition and can reflect how individuals adjust leukocyte investment under differing infection, inflammatory, and physiological contexts [14,16–18]. Diet may influence these leukocyte traits indirectly through nutritional state, physiological stress, and exposure to pathogens or environmental stressors, although such relationships are probably species- and context-dependent [1,4,14,19]. Because species can differ in dietary composition and exposure to plant-derived compounds, variation in trophic ecology may be associated with multiple physiological dimensions in wild birds [9–11].

To evaluate such diet-related variation under natural conditions, stable isotope analysis offers a useful framework for quantifying trophic ecology in wild birds [20–22]. In consumer tissues, δ^15^N values are commonly used as a proxy for trophic position, whereas δ^13^C values can help distinguish among basal carbon sources or foraging habitats depending on ecological context [20–22]. Variation in δ^13^C–δ^15^N space can also be used to estimate isotopic niche width, which provides an integrative measure of dietary breadth or resource-use variation within species [20,23–25]. These isotope-based descriptors make it possible to evaluate whether trophic position or niche breadth is more closely associated with physiological variation in free-living birds [2,21].

Although previous studies have linked diet or trophic ecology to specific components of physiological state—including oxidative balance, immune-related traits, and metabolic markers—these investigations have often focused on single physiological axes or particular ecological contexts [1,2,4,19]. Consequently, less is known about how trophic position and isotopic niche breadth relate to coordinated physiological variation across multiple functional pathways in wild bird communities [1,2,21]. Trophic position and isotopic niche breadth capture different aspects of trophic ecology. Trophic position reflects the average level at which individuals feed within the local food web [20–22] and may therefore be more directly associated with broad differences in resource type, nutrient intake, and exposure to dietary compounds [2,9–11,21]. By contrast, isotopic niche breadth reflects variation in resource use within species [20,23–25] and may be associated more closely with dietary diversity and physiological flexibility than with the predominant feeding ecology [21,25]. Both dimensions could therefore be related to physiological variation, but potentially through different mechanisms [2,21].

In this study, we examined whether trophic position and isotopic niche breadth are associated with physiological variation in three sympatric passerine species from central Chile that represent distinct trophic categories. We captured *Rhopospina fruticeti*, *Zonotrichia capensis*, and *Pteroptochos megapodius* during the non-breeding season and measured blood δ^13^C and δ^15^N values together with total and differential leukocyte counts, antioxidant capacity, uric acid, and enzymatic markers of oxidative metabolism, glutathione-dependent detoxification and redox regulation, and plasma esterase-associated detoxification. We then tested whether trophic position and isotopic niche breadth were associated with multivariate physiological differentiation among species. We predicted that both trophic position and isotopic niche breadth would be associated with physiological variation, but that trophic position might show a more direct association with metabolic and enzyme-based markers. We also expected leukocyte profiles to covary with these traits in ways consistent with physiological stress and resource allocation. Given the limited number of species examined, our study was designed as an exploratory interspecific comparison rather than a phylogenetically informed comparative analysis.

## 2. Material and methods

### 2.1. Model species and bird capture

We studied three resident passerine species from the sclerophyllous forest of central Chile: *R. fruticeti* (*n* = 20), *Z. capensis* (*n* = 20), and *P. megapodius* (*n* = 20). *P. megapodius* is a ground-dwelling insectivore that forages in the understory, *R. fruticeti* is primarily granivorous and inhabits open shrublands, and *Z. capensis* is an omnivore typically associated with vegetation edges and clearings [26].

Birds were captured in the Metropolitan Region of Chile at the Estación Agronómica Experimental Germán Greve Silva, Rinconada de Maipú (33°30′ S, 70°54′ W), using mist nets deployed between 09.00 and 16.00 during the non-breeding season (June–August) of 2024. By restricting sampling to the non-breeding season, we reduced potential variation associated with reproductive condition. Sex and age were not determined, so their contribution to the observed variation could not be evaluated**.**

Nets were continuously monitored, and all birds were bled within 1–5 min of capture. Immediately after capture, we collected three blood samples. First, approximately 100–200 µl of blood was collected from the brachial vein using heparinized microcapillary tubes for enzymatic assays. Second, a smaller blood sample (approx. 5 µl) was placed directly onto a microscope slide to prepare a blood smear for leukocyte analyses. Third, approximately 50 µl of whole blood was transferred onto a glass slide and air-dried in the field for stable isotope analysis. All birds were released at the capture site immediately after sampling.

Blood samples were transported in insulated containers at approximately 4°C for less than 1 h to the laboratory, located 60 km from the study site. Samples were centrifuged at 10 000 r.p.m. and 4°C for 10 min to separate plasma from red blood cells. Plasma aliquots and red blood cells were stored at −80°C until biochemical analyses were performed. Because blood volume was limited, not all measurements could be obtained for every individual, and sample size therefore differed among variables.

### 2.2. Leukocyte counts

Blood smears were air-dried, fixed in absolute methanol for 2 min, and stained with May–Grünwald–Giemsa [27]. Slides were examined under a light microscope at 1000× magnification with oil immersion. To estimate total white blood cell count, we counted leukocytes in 10 random non-overlapping fields within the monolayer zone of each smear [28]. Estimated white blood cell count (cells µl^−1^) was calculated as the mean number of leukocytes per field multiplied by 2000 [29].

We then performed a differential leukocyte count by classifying 100 leukocytes per smear as heterophils, lymphocytes, eosinophils, basophils, or monocytes based on morphology [29]. The H:L ratio was calculated as the number of heterophils divided by the number of lymphocytes [13].

During leukocyte counts, blood smears were also opportunistically screened for blood-borne microfilariae compatible with Onchocercidae. Individuals were recorded as positive when at least one microfilaria was detected in the examined fields. Because this screening was limited to the fields evaluated during leukocyte quantification, these observations were treated as qualitative detections rather than as a robust estimate of parasite prevalence or infection intensity [30].

### 2.3. Total antioxidant capacity and uric acid

We quantified non-enzymatic total antioxidant capacity (TAC) using the colorimetric method described by Sabat *et al.* [31], based on Apak *et al.* [32]. This assay measures the capacity of plasma antioxidants to reduce Cu²⁺ to Cu¹⁺, which is then detected by reaction with neocuproine and quantified spectrophotometrically at 450 nm. TAC provides an integrative estimate of the combined action of low-molecular-weight antioxidants in plasma.

Plasma uric acid concentration was measured using an enzymatic-colorimetric kit (Valtek Diagnostics, Chile) based on the uricase/peroxidase method. Because uric acid contributes substantially to TAC in birds, it was measured separately to aid interpretation of variation in antioxidant capacity [33].

### 2.4. Enzymatic activity of red blood cells

Red blood cells were homogenized in 100 mM potassium phosphate buffer (pH 7.2) containing 2 mM EDTA. Citrate synthase (CS) activity was measured at 412 nm and 25°C using a DTNB-coupled assay [34]. Activity was expressed as nmol min^−1^ mg protein^−1^.

Glutathione reductase (GR) activity was measured at 340 nm and 25°C by monitoring NADPH oxidation [35]. Activity was expressed as nmol min^−1^ mg protein^−1^.

Glutathione S-transferase (GST) activity was measured at 340 nm and 25°C based on conjugation of reduced glutathione with 1-chloro-2,4-dinitrobenzene [36]. Activity was expressed as nmol min^−1^ mg protein^−1^.

Protein concentration was determined using a bicinchoninic acid assay (Pierce BCA Protein Assay Kit, Thermo Fisher Scientific). All assays were performed with a Thermo Scientific Multiskan SkyHigh spectrophotometer.

### 2.5. Enzymatic activity of plasma

Acetylcholinesterase (AChE) activity in plasma was measured at 412 nm and 25°C following Wheelock *et al.* [12], based on Ellman *et al.* [37]. Activity was expressed as nmol min^−1^ mg protein^−1^.

Carboxylesterase (CbE) activity in plasma was measured at 405 nm and 25°C using a modified end-point assay based on Sánchez-Hernández *et al.* [38] and Chanda *et al.* [39], with 4-nitrophenyl acetate as substrate. Activity was expressed as nmol min^−1^ mg protein^−1^.

To summarize relative variation in plasma esterase activities, we calculated an esterase index as the ratio of CbE to AChE activity. In the present study, this ratio was used as a comparative indicator of detoxification-related physiological variation across individuals. Because CbEs can participate in the hydrolysis of some pesticide compounds, this index may also be useful in future work evaluating pesticide-related detoxification and is used here as a comparative indicator of detoxification-related physiological variation rather than as a pesticide-specific endpoint [12,40]. All assays were performed with a Thermo Scientific Multiskan SkyHigh spectrophotometer.

### 2.6. Stable isotope analysis

Whole-blood samples for stable isotope analysis were dried for an additional 48 h at 60°C. Dried material was scraped from slides using a sterile scalpel and transferred to microcentrifuge tubes. Approximately 0.5 mg of dried blood was weighed into tin capsules for stable carbon (δ^13^C) and nitrogen (δ^15^N) isotope analysis.

δ ^13^C and δ^15^N values were measured with a Costech 4010 elemental analyser coupled to a Thermo Scientific Delta V Plus isotope-ratio mass spectrometer at the Centre for Stable Isotopes, University of New Mexico (Albuquerque, NM). Isotope ratios are expressed in standard *δ* notation as

$$\begin{array}{r} \delta X=\left[\left(\frac{R_{sample}}{R_{standard}}\right)-1\right]\times 1000, \end{array}$$

where *X* is ^13^C or ^15^N, and *R* is the corresponding heavy-to-light isotope ratio. Vienna Pee Dee Belemnite and atmospheric N₂ were used as the standards for carbon and nitrogen, respectively.

Trophic level (TL) was estimated using diet-specific trophic discrimination factors for whole blood [41]: 3.5‰ for *R. fruticeti*, 2.2‰ for *Z. capensis*, and 2.3‰ for *P. megapodius*. Estimated TL was calculated as

$$\begin{array}{r} \text{TL}=1+\frac{\delta^{15}N_{tissue}-\delta^{15}N_{baseline}}{\Delta^{15}N}, \end{array}$$

where Δ^15^N is the species-appropriate discrimination factor. Baseline δ^15^N was estimated from leaf samples of nine plant species collected at the study site during winter. Mean baseline δ^15^N was 3.5 ± 4.0‰, consistent with previous values reported for the same locality [7,42]. Higher TL estimates indicate greater ^15^N enrichment relative to the δ^15^N composition of plants at the base of local food webs [20,43,44].

### 2.7. Statistical analysis

Before conducting the ANOVAs, model assumptions were evaluated using residual diagnostic plots, Shapiro–Wilk tests for normality, and Levene's tests for homogeneity of variance. No transformation was required for most variables. For variables that did not meet the assumptions of the standard ANOVA, one-way ANOVAs were conducted on rank-transformed data. We used one-way ANOVA to test for differences among species in physiological, biochemical, and isotopic variables. To account for multiple testing across the 11 physiological and ecological traits and control the false discovery rate without unduly reducing statistical power, we applied the Benjamini–Hochberg procedure with a false discovery rate threshold set to *Q* = 0.05 [45]. For variables showing significant overall differences among species, *post hoc* pairwise comparisons were conducted using Tukey's honestly significant difference (HSD) test. Spearman's rank correlation was used to assess the association between TAC and uric acid concentration, as well as other bivariate relationships evaluated in the Results.

To compare isotopic niche width among species, we calculated small-sample corrected standard ellipse areas (SEA_C_) and Bayesian standard ellipse areas (SEA_B_) using the SIBER framework [23]. Bayesian ellipses were estimated from 10 000 Markov chain Monte Carlo iterations to obtain posterior distributions, credibility intervals, and pairwise probabilities that one species had a larger isotopic niche than another. Graphical niche representations were based on SEA_C_.

To summarize multivariate physiological variation, we used principal component analysis (PCA) with varimax rotation on standardized physiological variables. Because complete multivariate analysis required a full data matrix, missing values caused by limited sample volume were handled by stochastic imputation performed separately within species [46,47]. For each variable, missing values were replaced by random draws from a normal distribution parametrized by the species-specific mean and standard deviation estimated from observed values. PCA scores from the first three components were then extracted and analysed by ANOVA to test for interspecific differences in multivariate physiological structure. ANOVAs on individual variables tested species for differences among species in specific physiological traits, whereas ANOVAs on PCA scores tested species for differences among species in overall multivariate physiological structure [46,47]. All analyses were conducted in R version 4.4.1, and statistical significance was evaluated at *α* = 0.05.

## 3. Results

### 3.1. Stable isotope variation and trophic structure

Stable isotope analyses revealed clear interspecific differences in trophic structure among the three passerine species. *P. megapodius* had the highest δ^13^C and δ^15^N values and the highest estimated TL; *Z. capensis* occupied an intermediate position, and *R. fruticeti* had TL estimates consistent with a primary consumer (figure 1A; supplementary material, table S2). One-way ANOVAs detected significant differences among species for δ^13^C (*F*_(2, 43)_ = 10.76, *p* < 0.001), δ^15^N (*F*_(2, 43)_ = 3.68, *p* = 0.038), and estimated TL (*F*_(2, 43)_ = 16.75, *p* < 0.001). *Post hoc* Fisher tests indicated that *P. megapodius* occupied a significantly higher trophic position than *R. fruticeti*, with *Z. capensis* showing intermediate values.

**Figure 1.**
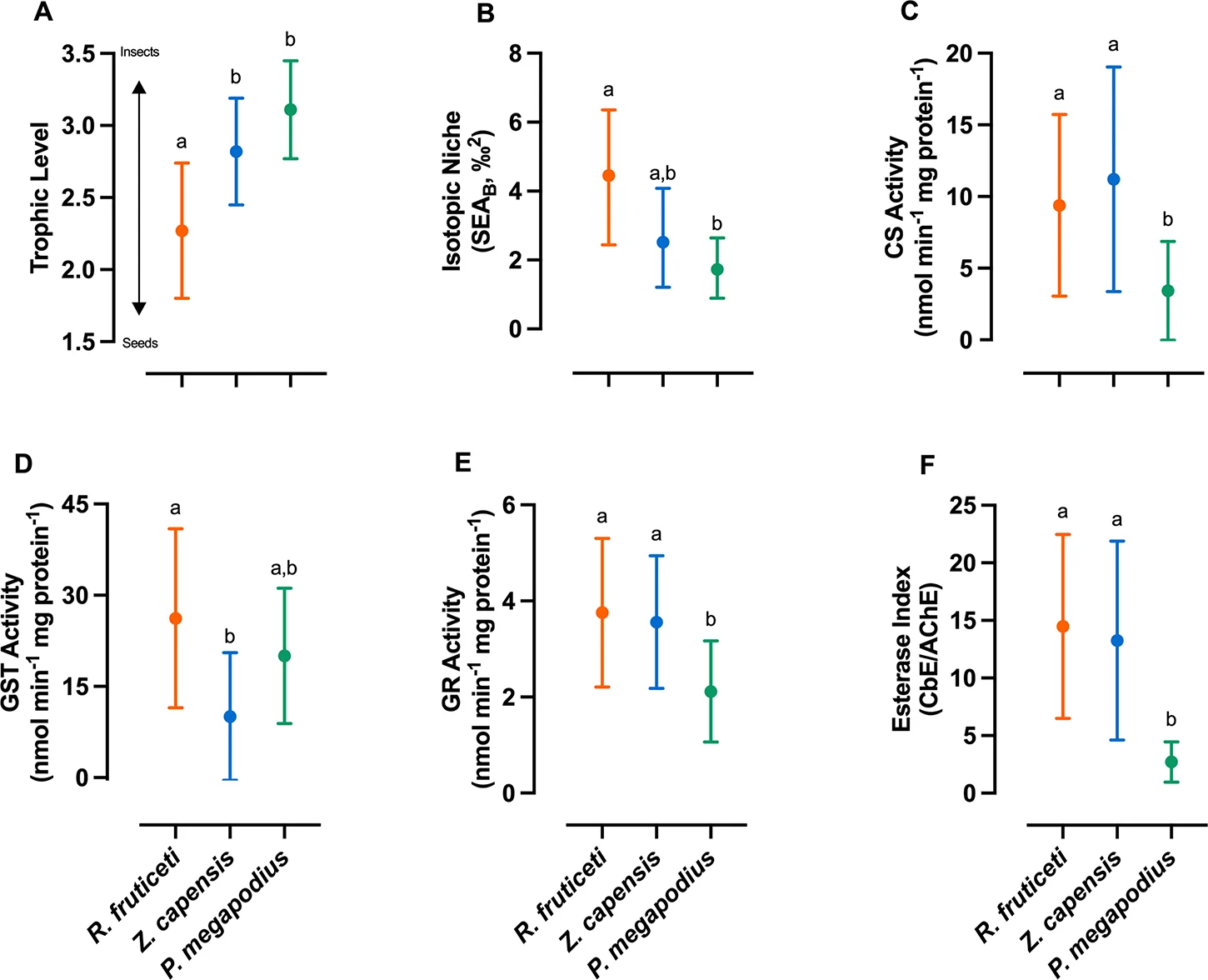
Physiological and isotopic traits in three sympatric passerine species, including (A) estimated TL (*n* = 46), (B) SEA_B_ (*n* = 46), (C) CS activity (*n* = 36), (D) GST activity (*n* = 39), (E) GR activity (*n* = 37), and (F) esterase index (CbE/AChE; *n* = 43). Higher TL values indicate feeding at a higher position in the local food web. Circles denote means and error bars represent s.e. for TL and enzymatic activities, whereas SEA_B_ values are shown as medians with 95% credible intervals. Different letters indicate significant differences (*p* < 0.05) among species based on one-way ANOVA followed by Tukey's (HSD) *post hoc* tests for TL and enzymatic activities. For SEA_B_, differences were assessed from posterior probabilities generated by Bayesian iterations (see §2 for details).

In contrast with trophic position, isotopic niche width did not follow the same gradient. SEA_B_ and SEA_C_ indicated that *R. fruticeti* had the broadest isotopic niche, which was significantly larger than that of *P. megapodius* and marginally larger than that of *Z. capensis* (figure 1B; supplementary material, table S3). *P. megapodius* exhibited the narrowest isotopic niche despite occupying the highest trophic position, whereas *Z. capensis* showed intermediate isotopic niche width (figure 1B; supplementary material, figure S1).

### 3.2. Metabolic and enzyme-based variables

Several enzymatic markers differed significantly among species (figure 1). CS activity differed among species (*F*_(2, 33)_ = 5.07, *p* = 0.012; figure 1C). *P. megapodius* had lower CS activity than *Z. capensis* (*p* = 0.012) and *R. fruticeti* (*p* = 0.040), whereas *Z. capensis* and *R. fruticeti* did not differ (*p* = 0.862).

GST activity also differed among species (*F*_(2, 36)_ = 6.21, *p* = 0.005; figure 1D). *R. fruticeti* showed higher GST activity than *Z. capensis* (*p* < 0.001), whereas *P. megapodius* had intermediate values and did not differ significantly from either species (*p* > 0.05).

GR activity differed among species (*F*_(2, 34)_ = 3.66, *p* = 0.036; figure 1E). *P. megapodius* had lower GR activity than *Z. capensis* (*p* = 0.026) and *R. fruticeti* (*p* = 0.017), whereas *Z. capensis* and *R. fruticeti* did not differ (*p* = 0.888).

The esterase index (CbE/AChE) also differed among species (*F*_(2, 40)_ = 26.55, *p* < 0.0001; figure 1F). *Z. capensis* had higher values than *P. megapodius* (*p* < 0.001), and *R. fruticeti* also had higher values than *P. megapodius* (*p* < 0.001), whereas *Z. capensis* and *R. fruticeti* did not differ (*p* = 0.970).

### 3.3. Leukocyte variables and opportunistic parasite detections

Total and differential leukocyte counts differed significantly among species. Mean H:L ratios varied among species (*F*_(2, 39)_ = 8.095, *p* = 0.001; figure 2A) and were significantly higher in *P. megapodius* than in both *Z. capensis* (*p* = 0.014) and *R. fruticeti* (*p* = 0.002), whereas *Z. capensis* and *R. fruticeti* did not differ from one another (*p* = 0.808). Total white blood cell counts also varied among species (*F*_(2, 39)_ = 4.82, *p* = 0.013; figure 2B). *R. fruticeti* had significantly higher total leukocyte counts than both *P. megapodius* (*p* = 0.018) and *Z. capensis* (*p* = 0.05), whereas *P. megapodius* and *Z. capensis* did not differ (*p* = 0.945).

**Figure 2.**
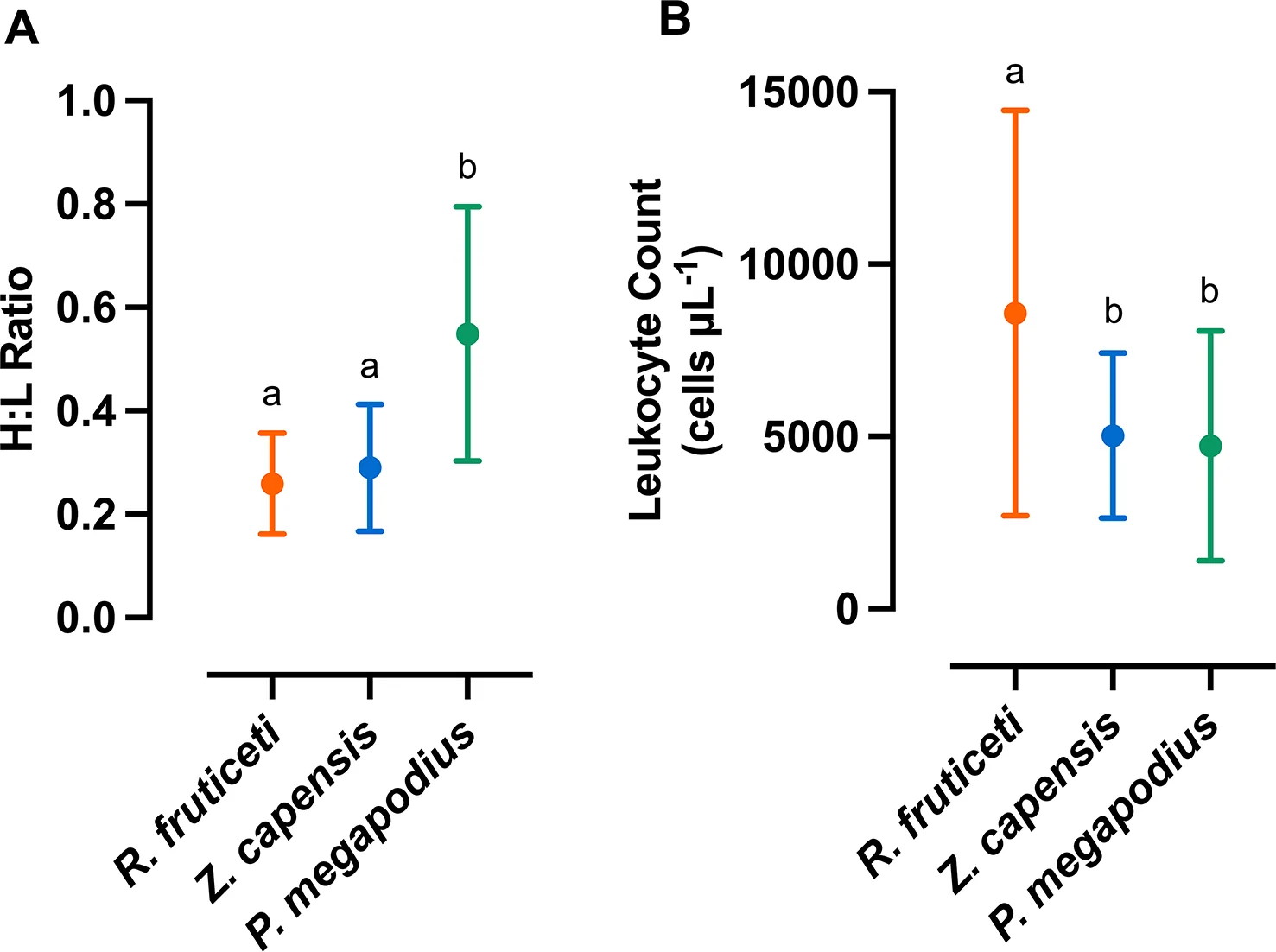
Mean (A) H:L ratios (*n* = 42) and (B) estimated total white blood cell counts (*n* = 42) in three sympatric passerine species. Error bars represent s.e. Different letters indicate significant differences (*p* < 0.05) among species based on one-way ANOVA followed by Tukey's (HSD) *post hoc* tests.

During opportunistic screening of blood smears performed in the course of Leukocyte counts, microfilariae compatible with Onchocercidae were detected in 2 of 14 examined *P. megapodius* smears. No microfilariae were detected in the smears examined from the other two species. Because screening effort was limited, these observations are reported as qualitative detections only.

### 3.4. Antioxidant measures

Systemic antioxidant measures did not differ significantly among species (figure 3). Neither TAC (*F*_(2, 47)_ = 0.07, *p* = 0.927; figure 3A) nor uric acid concentration (*F*_(2, 34)_ = 1.24, *p* = 0.301; figure 3B) varied among species.

**Figure 3.**
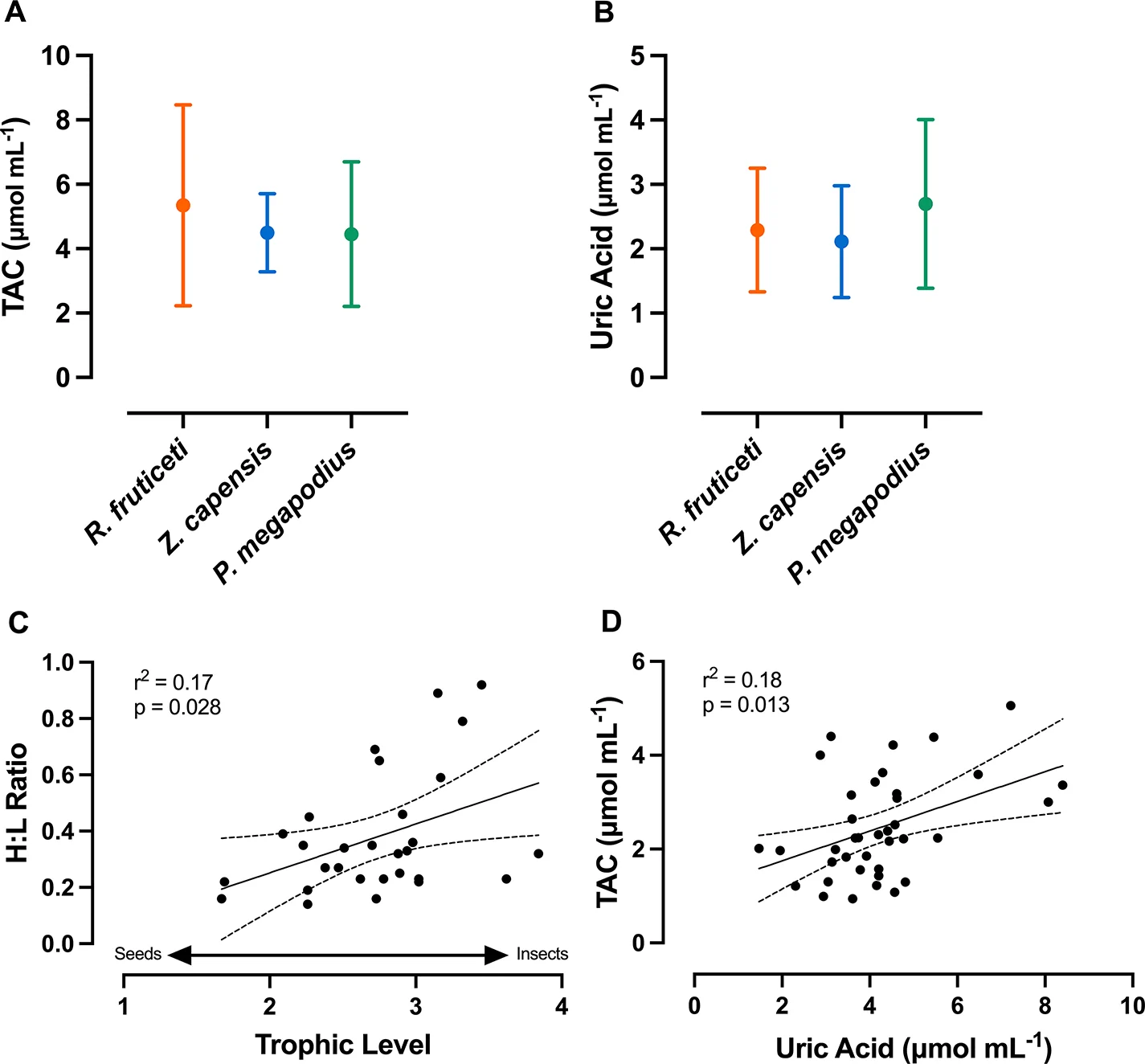
Mean (A) TAC (*n* = 50) and (B) uric acid concentration (µmol l^−1^; *n* = 37) in three sympatric passerine species. Error bars represent s.e. No significant interspecific differences were detected for TAC or uric acid concentration. (C) The positive correlation between H:L ratio and estimated TL across all species (*r* = 0.410, *p* = 0.028; *n* = 29). Higher TL values indicate feeding at a higher position in the local food web. (D) The positive association between TAC and plasma uric acid across individuals (*n* = 37).

Correlation analyses revealed selective associations among ecological and physiological variables. TL was positively correlated with H:L ratio (*r* = 0.410, *p* = 0.028; figure 3C). CS activity was negatively correlated with H:L ratio (*r* = −0.369, *p* = 0.027). GST activity was not significantly correlated with TL (*r* = 0.214, *p* = 0.352). GR activity was positively correlated with CS activity (*r* = 0.290, *p* = 0.048). Uric acid concentration was positively correlated with TAC (*r* = 0.364, *p* = 0.013; figure 3D).

### 3.5. Multivariate analysis

To summarize covariation among physiological variables, we performed PCA with varimax rotation on standardized physiological traits after species-specific stochastic imputation of missing values. The first three components had eigenvalues greater than 1 and together explained 65.0% of the total variance (PC1: 27.0%, PC2: 19.8%, PC3: 18.2%; supplementary material, table S4).

PC1 showed strong positive loadings for CS activity and the esterase index. PC2 was defined primarily by GST activity and H:L ratio. PC3 loaded positively on TAC and uric acid concentration. GR showed high uniqueness and did not contribute strongly to the multivariate structure (supplementary material, table S4).

ANOVAs on individual PCA scores revealed significant interspecific differences for PC1 (*F*_(2, 50)_ = 24.954, *p* < 0.001) and PC2 (*F*_(2, 50)_ = 5.329, *p* = 0.008), whereas PC3 did not differ among species (*F*_(2, 50)_ = 0.574, *p* = 0.567; figure 4). For PC1, *P. megapodius* differed significantly from both *Z. capensis* (*p* < 0.001) and *R. fruticeti* (*p* < 0.001), whereas *Z. capensis* and *R. fruticeti* did not differ (*p* = 0.980). For PC2, *Z. capensis* differed significantly from *P. megapodius* (*p* = 0.006), whereas *Z. capensis* did not differ from *R. fruticeti* (*p* = 0.123) and *P. megapodius* did not differ from *R. fruticeti* (*p* = 0.423). No pairwise differences were detected for PC3.

**Figure 4.**
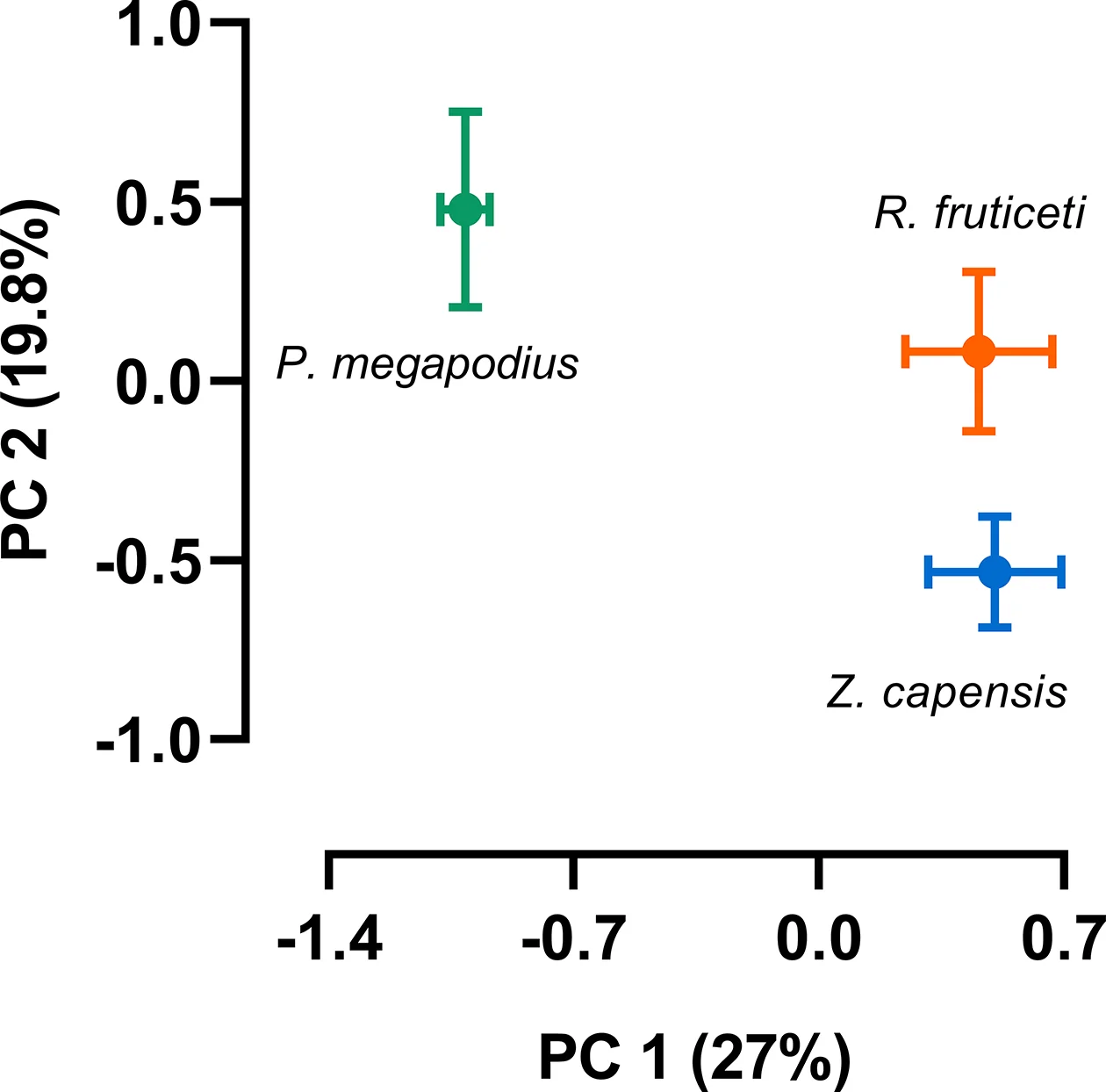
PCA of physiological variables among three sympatric passerines showing mean scores (±s.e.) for PC1 and PC2 (*n* = 53). PC1 was associated primarily with CS activity and the esterase index, whereas PC2 was associated with glutathione S-transferase activity and H:L ratio. Both PC1 and PC2 differed significantly among species. Together, these two components explained 46.8% of total variance (PC1 = 27.0%, PC2 = 19.8%). PC3, associated with TAC and uric acid concentration, did not differ among species and is not shown.

Across the 11 univariate ANOVAs, nine traits showed significant interspecific differences after Benjamini–Hochberg FDR correction (*Q* = 0.05): δ^13^C, δ^15^N, estimated trophic position, CS, GST, GR, the esterase index (CbE/AChE), H:L ratio, and total white blood cell count. By contrast, TAC and uric acid concentrations did not differ significantly among species, either before or after correction (see supplementary material, table S1).

## 4. Discussion

Our results suggest that physiological differentiation in this sympatric passerine assemblage was more closely associated with trophic position than with isotopic niche breadth. Species differed clearly in trophic position and in several physiological traits, whereas isotopic niche width showed weaker correspondence with physiological variation. Across species, TAC and uric acid remained broadly similar and were positively correlated, suggesting that baseline non-enzymatic antioxidant buffering was comparatively conserved [33]. By contrast, metabolic and enzyme-based variables contributed more strongly to interspecific physiological differentiation.

Leukocyte variables also differed among species, indicating interspecific differences in leukocyte profiles. In birds, elevated H:L ratios are commonly interpreted as indicators of stress-associated shifts in investment among leukocyte classes [13,15], whereas total leukocyte counts capture a distinct dimension of physiological variation under differing infection, inflammatory, and nutritional contexts [14,17,18]. Together, these patterns are consistent with interspecific differences in physiological stress and leukocyte profile potentially associated with trophic ecology [13,14].

Within this framework, *P. megapodius*, the species occupying the highest trophic position, showed higher H:L ratios together with lower CS activity. This pattern suggests an association between trophic ecology, oxidative metabolism, and variation in leukocyte profile among species [13]. CS is widely used as a proxy of oxidative metabolic capacity in ecological physiology, and avian studies increasingly interpret blood- or tissue-based metabolic enzymes in relation to whole-organism energetics [48–50]. The negative association between CS activity and H:L ratio, therefore, points to a linkage between metabolic phenotype and leukocyte profile, although the mechanism underlying this association remains unresolved [48,50]. More direct tests of whole-organism metabolic performance, endocrine stress physiology, and additional immune traits would be needed to determine whether these variables are connected through energetic trade-offs [51].

A further possibility is that differences in parasite exposure contributed to some of the leukocyte variation observed among species. Microfilariae compatible with Onchocercidae were detected only in a small number of *P. megapodius* smears during opportunistic screening performed in the course of leukocyte counts. Although screening effort was limited, this observation raises the possibility that species-level differences in parasite exposure or burden, potentially associated with trophic ecology or microhabitat use, could have contributed to variation in leukocyte profiles [30]. Dedicated parasite screening would be required to evaluate that possibility more directly.

At the same time, the interspecific pattern was not captured simply by variation in isotopic niche breadth. *P. megapodius*, which occupied the highest trophic position, showed higher H:L ratios together with lower CS and GR activity. Within this assemblage, that combination is consistent with a physiological profile characterized by higher stress-associated leukocyte redistribution and lower oxidative metabolic and glutathione-recycling activity. By contrast, *R. fruticeti* showed relatively high glutathione-related and esterase-associated traits despite occupying the lowest trophic position, whereas *Z. capensis* generally occupied an intermediate trophic position without showing uniformly intermediate physiological values. Together, these patterns suggest that species-specific patterns in physiological traits were associated with trophic position.

Glutathione-related and plasma esterase variables showed particularly clear species-level differentiation. GST, GR, and the esterase index varied among species, indicating interspecific differences in conjugative metabolism, redox recycling, and detoxification-related physiological profiles [12,35,52,53]. GST plays a central role in xenobiotic conjugation and also interfaces with antioxidant defence through glutathione-dependent metabolism [52,53], whereas GR contributes to maintaining glutathione redox cycling [35]. In this context, the relatively high glutathione-related and esterase-associated values observed in the granivorous species occupying lower trophic position may be consistent with differences in dietary composition, including variation in exposure to plant-derived compounds such as secondary metabolites [9–11]. Variation in these enzymatic profiles may therefore reflect differences in resource composition, lineage-specific physiology, or other ecological factors.

The multivariate analysis supports this interpretation. PCA indicated that the main axis of differentiation was driven primarily by CS activity and the esterase index, consistent with a metabolic–detoxification-related dimension of variation. A secondary axis was associated with GST activity and leukocyte profile variables, pointing to coordinated variation between glutathione-linked metabolism and leukocyte profile structure. This interpretation is consistent with broader ideas of phenotypic integration [54] and with recent evidence from passerines in central Chile showing that trophic and immune-related traits can form structured multivariate functional gradients while differing in seasonal flexibility [55]. By contrast, the component defined by TAC and uric acid did not differ among species, reinforcing the view that baseline non-enzymatic antioxidant buffering was comparatively conserved [33].

The comparative scope of this study defines the level at which these patterns can be interpreted. We examined only three passerine species, each with a modest sample size, and these species cannot be treated as independent evolutionary replicates; also, our study design does not allow for a phylogenetically informed comparative analysis [1]. In particular, *P. megapodius* is a sub-oscine, whereas *R. fruticeti* and *Z. capensis* are oscines and more closely related. Thus, the closer relationship between the two oscine species, as well as the consistent differences in ecological and physiological metrics we measured between *P. megapodius* and the other two species, could reflect phylogenetic relatedness as well as ecological differentiation. We cannot determine whether the observed interspecific patterns reflect ecological associations with trophic position or lineage-specific traits, or a combination of both. In addition, sex and age were not included as explanatory variables, and their contribution to the observed variation cannot be excluded. The study should therefore be regarded as a comparative analysis among species along a trophic spectrum that identifies interspecific associations and generates testable hypotheses, rather than as a definitive test of adaptive variation. Furthermore, the sympatric nature of our study design helps reduce major sources of environmental heterogeneity associated with climate, season, and habitat context [24,34], but it does not eliminate phylogenetic non-independence. Broader taxonomic sampling, including multiple independent lineages spanning comparable trophic positions, together with formal phylogenetic analyses, will be required to assess the generality and evolutionary basis of these patterns.

Overall, our results indicate that trophic position was more closely associated than isotopic niche breadth with coordinated physiological variation in this passerine community. Physiological variation was expressed most clearly in metabolic and enzyme-based markers, whereas leukocyte profiles added a complementary dimension related to interspecific differences in leukocyte allocation [13,14]. Future work combining fuller immune assays, dedicated parasite screening, direct characterization of dietary compounds, broader taxonomic sampling, and formal phylogenetic comparisons will help clarify the generality and mechanistic basis of these patterns.

## Supplementary Material



## Data Availability

The datasets generated and analysed are available online [56]. Supplementary material is available online [57].
