# Supplementary material for: Trophic position structures physiological trade-offs between metabolic and detoxification pathways in sympatric passerines
Source: R Soc Open Sci. 2026 Oct 7;13(10):261499. doi: 10.1098/rsos.261499 (PMC13641901; doi:10.1098/rsos.261499)
Supplement: Supplementary file 1 [file rsos.261499.zip › rsos.261499/rsos261499_si_001.docx]

**Supplementary Material**

**Table S1.** Benjamini–Hochberg false discovery rate (FDR) correction for multiple comparisons across 11 physiological and ecological traits. Original p-values from univariate ANOVAs testing interspecific differences were ranked in ascending order. The Benjamini–Hochberg critical value was calculated for each trait using the formula BH = (i/m)Q, where m is the total number of tests (m = 11) and Q is the prespecified false discovery rate (Q = 0.05). Significance was determined by identifying the highest rank for which the original p-value was less than or equal to its corresponding critical value. In this analysis, the highest qualifying rank was rank 9, corresponding to δ^15^N; traits with ranks 1–9 were considered statistically significant after correction.

| **Rank (i)** | **Physiological/Ecological Trait** | **p-value** | **BH Critical Value** | **Significance** |
| --- | --- | --- | --- | --- |
| 1 | Esterase index (CbE/AChE) | <0.0001 | 0.005 | Yes |
| 2 | Trophic position | <0.001 | 0.009 | Yes |
| 3 | δ^13^C | <0.001 | 0.014 | Yes |
| 4 | H:L ratio | 0.001 | 0.018 | Yes |
| 5 | GST activity | 0.005 | 0.023 | Yes |
| 6 | Total WBC | 0.013 | 0.027 | Yes |
| 7 | CS activity | 0.012 | 0.032 | Yes |
| 8 | GR activity | 0.036 | 0.036 | Yes |
| 9 | δ^15^N | 0.038 | 0.041 | Yes |
| 10 | Uric acid concentration | 0.301 | 0.045 | No |
| 11 | Total antioxidant capacity (TAC) | 0.900 | 0.050 | No |

## Reference

[45] Y. Benjamini and Y. Hochberg, “Controlling the false discovery rate: a practical and powerful approach to multiple testing,” 1995. doi: [10.1111/J.2517-6161.1995.TB02031.X](https://doi.org/10.1111/J.2517-6161.1995.TB02031.X)

**Table S2.** Mean values and standard deviation (± SD) of body mass (mb), blood carbon (^13^C) and nitrogen (^15^N) isotope values, and trophic level estimates. Different superscript letters indicate significant differences after Fisher’s post-hoc test.

|  | Species | | | Statistics | |
| --- | --- | --- | --- | --- | --- |
| Variable | *Z. capensis* | *P. megapodius* | *R. fruticeti* | F (df) | p |
| N* | 12 | 15 | 18 | - | - |
| Body  Mass (g) | 21.3 ± 1.8 | 127.0 ± 19.6 | 38.6 ± 3.7 | 333.41 (2, 24.9) | **< 0.001** |
| δ^13^C | -26.2 ± 0.9^a^ | -25.1 ± 0.5^b^ | -26.6 ± 0.9^a^ | 18.21 (2, 24.5) | **< 0.001** |
| δ^15^N | 7.4 ± 0.8^ab^ | 8.2 ± 0.9^b^ | 7.2 ± 1. 3^a^ | 3.68 (2, 28.5) | **0.038** |
| Trophic Level | 2.8 ± 0.3^a^ | 3.1 ± 0.4^a^ | 2.1 ± 0.4^b^ | 28.03 (2, 43) | **< 0.001** |

**Table S3**. Standard ellipse area (SEA), standard ellipse area corrected for small sample sizes (SEA_C_), and Bayesian standard ellipse area (SEA_B_), expressed in ‰², for three passerines (*Z. capensis*, *P. megapodius*, and *R. fruticeti*) inhabiting central Chile. Significant differences among the size of ellipse areas are indicated with asterisks.

| Group/Species | SEA | SEA_C_ | SEA_B_ | P: G > G1 | P: G > G2 |
| --- | --- | --- | --- | --- | --- |
| G1.  *Z. capensis* | 2.293 | 2.522 | 2.515 | **-** | 0.167 |
| G2. *P.megapodius* | 1.605 | 1.729 | 1.731 | 0.833 | **-** |
| G3.  *R. fruticeti* | 4.216 | 4.464 | 4.452 | 0.938 | **0.997*** |

**Table S4**. Principal component axis derived of analyses of physiological features in three passerine species from central Chile.

| **Component** | | | |
| --- | --- | --- | --- |
| **Factors** | **PC 1** | **PC 2** | **PC 3** |
| **Citrate Synthase (CS)** | **0.815** |  |  |
| **Esterase Index** | **0.832** |  |  |
| **Glutathione s-transferase (GST)** |  | **0.810** |  |
| **Heterophil:Leukocyte ratio (H:L)** |  | **0.668** |  |
| **Total antioxidant capacity (TAC)** |  |  | **0.852** |
| **Uric Acid (UA)** |  |  | **0.702** |
| **Glutathione reductase (GR)** | 0.449 |  |  |
| **Explained Variance (%)** | 27.0 | 19.8 | 18.2 |
| **Cumulative Variance (%)** | 27.0 | 46.8 | 65.0 |

Note: Loadings > |0.5| represent primary component membership. PCA1 = metabolic-enzymatic ; PCA2 = immune-detoxification ; PCA3 = non-antioxidant capacity. Varimax rotation applied.

**Figure S1.** Isotopic niches of three sympatric passerine species from central chilean sclerophyllous forest. Standard ellipse areas corrected for small sample sizes (SEAc, 40% probability contours in δ¹³C-δ¹⁵N space) represent relative dietary breadth and resource use. *Rhopospina fruticeti* (yellow, granivorous), *Pteroptochos megapodius* (green, insectivorous), and *Zonotrichia capensis* (blue, omnivorous) were field collected in winter 2024. Ellipse separation indicates trophic niche partitioning, with *P. megapodius* occupying the highest δ¹⁵N position.
